# Bioinformatic analysis identifying FGF1 gene as a new prognostic indicator in clear cell Renal Cell Carcinoma

**DOI:** 10.1186/s12935-021-01917-9

**Published:** 2021-04-17

**Authors:** Xiaoqin Zhang, Ziyue Wang, Zixin Zeng, Ningning Shen, Bin Wang, Yaping Zhang, Honghong Shen, Wei Lu, Rong Wei, Wenxia Ma, Chen Wang

**Affiliations:** 1 Department of Pathology, The Second Hospital of ShanXi Medical University, ShanXi Province, No.382 WuYi Road, Tai Yuan, 030000 China; 2 Department of Pathology, The Second Clinical Medical College of ShanXi Medical University, ShanXi Province, Tai Yuan, China

**Keywords:** GEO database, Protein–protein interaction network (PPI), Clear cell renal cell carcinoma (ccRCC), FGF1 gene, PI3K-AKT signaling pathway, Molecular pathology

## Abstract

**Background:**

Clear cell renal cell carcinoma (ccRCC) has been the commonest renal cell carcinoma (RCC). Although the disease classification, diagnosis and targeted therapy of RCC has been increasingly evolving attributing to the rapid development of current molecular pathology, the current clinical treatment situation is still challenging considering the comprehensive and progressively developing nature of malignant cancer. The study is to identify more potential responsible genes during the development of ccRCC using bioinformatic analysis, thus aiding more precise interpretation of the disease

**Methods:**

Firstly, different cDNA expression profiles from Gene Expression Omnibus (GEO) online database were used to screen the abnormal differently expressed genes (DEGs) between ccRCC and normal renal tissues. Then, based on the protein–protein interaction network (PPI) of all DEGs, the module analysis was performed to scale down the potential genes, and further survival analysis assisted our proceeding to the next step for selecting a credible key gene. Thirdly, immunohistochemistry (IHC) and quantitative real-time PCR (QPCR) were conducted to validate the expression change of the key gene in ccRCC comparing to normal tissues, meanwhile the prognostic value was verified using TCGA clinical data. Lastly, the potential biological function of the gene and signaling mechanism of gene regulating ccRCC development was preliminary explored.

**Results:**

Four cDNA expression profiles were picked from GEO database based on the number of containing sample cases, and a total of 192 DEGs, including 39 up-regulated and 153 down-regulated genes were shared in four profiles. Based on the DEGs PPI network, four function modules were identified highlighting a FGF1 gene involving PI3K-AKT signaling pathway which was shared in 3/4 modules. Further, both the IHC performed with ccRCC tissue microarray which contained 104 local samples and QPCR conducted using 30 different samples confirmed that FGF1 was aberrant lost in ccRCC. And Kaplan–Meier overall survival analysis revealed that FGF1 gene loss was related to worse ccRCC patients survival. Lastly, the pathological clinical features of FGF1 gene and the probable biological functions and signaling pathways it involved were analyzed using TCGA clinical data.

**Conclusions:**

Using bioinformatic analysis, we revealed that FGF1 expression was aberrant lost in ccRCC which statistical significantly correlated with patients overall survival, and the gene’s clinical features and potential biological functions were also explored. However, more detailed experiments and clinical trials are needed to support its potential drug-target role in clinical medical use.

**Supplementary Information:**

The online version contains supplementary material available at 10.1186/s12935-021-01917-9.

## Background

Rising from renal tubular epithelial cells, renal cell carcinoma has been a common malignant tumor, which ranks only second to bladder carcinoma in adults urinary tract malignant tumors [1]. And within RCC, 65 ~ 70% is clear cell renal cell carcinoma (ccRCC), which possess specific microscopic appearance other than the other RCC subtypes. Attributing to the rapid development of molecular pathology, the classification and diagnosis of renal cell carcinoma have been increasingly evolving [2, 3]. Currently, many molecular genetic abnormalities have been reported to exist in RCC, including chromosome number or structural abnormality, genes mutation, amplification or fusion genes resulting from chromosome translocation [4]. In ccRCC, the most classic molecular genetic characteristics are the changes of related genes on the short arm of chromosome 3 (3p), especially the VHL gene. The “first hit” of cancer usually comes from the change of VHL gene (gene mutation or promoter methylation), followed by "second hit"—3p chromosome deletion, which leads to tumor occurrence, and the 3p variation occurs in nearly 90% of ccRCC cases [5]. Besides the VHL gene, some other 3p gene variations have also been reported in ccRCC, for instance SETD2 [6] and BAP1 [7], whose mutation have been reported to be related with worse patients prognosis, as well as PBRM1 [8], which was associated with better patients survival.

As for the clinical cure methods, besides the traditional surgery and stereotactic body radiation therapy, great improvements have been taking place in molecular targeted therapies. At present, 13 drugs in 6 categories have been approved for metastatic ccRCC, including VEGFR, mTORC1, c-Met and FGFR inhibition, as well as cytokines and anti PD-1/PD-L1 immune checkpoint inhibitors [9–11]. These drugs have been showing promising curative effects and increasing the median patients survival time from 15 to 30 months in the past 10 years [4]. However, the curative effective obviously vary among different individuals indicating the heterogeneity in drug mechanisms, tumor molecular genetic changes and host immune situations. It is of great importance to keep identifying new potential prognostic biomarkers as well as probable drug targeting genes thus aiding more precise understanding of the disease.

Currently, with the gradual maturity and promotion of molecular pathological detection technologies, for instance tissue microarray, protein chip, next generation sequencing (NGS) and single cell sequencing which have been bringing in tremendous molecular data, it is more convenient for us to identify more potential disease-causing gene alterations and better understand the molecular basis of cancer development [12–15].

Gene Expression Omnibus(GEO) has been a widely used online cancer research database for providing high-throughput genes expression data submitted by research institutions all over the world. In the study, different GEO datasets were used to screen the differently expressed genes (DEGs) in ccRCC comparing to normal kidney tissues, followed by series of bioinformatic analysis, for instance protein–protein interacting (PPI) network construction, function modules analysis and Kaplan–Meier survival analysis to identify the key genes that potentially regulate ccRCC development. Further, local hospital patents samples were used to explore the potential clinical significance of the key gene. The results shall provide useful insights to the unearth of potential new prognostic biomarkers and drug targeting gene candidates for clinical ccRCC patients.

## Materials and methods

### Data source: cDNA expression profiles from GEO database

From GEO online database [16], we picked four ccRCC cDNA expression profiles GSE53757 [17], GSE53000 [18], GSE71963 [19] and GSE68417 [20] based on the sample number (only the profiles that contain at least 40 samples covering both cancer and normal tissues were considered). And of the four profiles, GSE53757 was based on agilent GPL570 platform [HG-U133_Plus_2] Affymetrix Human Genome U133 Plus 2.0 Array, containing 72 ccRCC and 72 kidney normal samples. And GSE53000 profile was based on agilent GPL6244 platform [HuGene-1_0-st] Affymetrix Human Gene 1.0 ST Array, containing 56 ccRCC samples and 6 kidney normal tissues. Meanwhile, GSE71963 was based on agilent GPL6480 platform Agilent-014850 Whole Human Genome Micro array 4×44K G4112F and contains 32 ccRCC and 16 normal kidney samples, as well as GSE68417 which was based on agilent GPL6244 platform [HuGene-1_0-st] Affymetrix Human Gene 1.0 ST Array, contains 29 ccRCC and 14 normal samples(Detailed information and accessing weblink in Additional file 1: Table S1).

### Screen the DEGs in ccRCC comparing to normal renal tissues

After the four cDNA expression profiles being downloaded from GEO database, GEO2R [21], which has been a widely used genes expression analyzing tool and commonly provided paired with GEO profiles online was used to screen the DEGs between ccRCC versus normal tissues. The criteria for DEGs identification were set as adjusted P value < 0.05 and |log2FC|≥ 2. Further, Venn diagram [22] was used to identify the DEGs that were shared in all four cDNA profiles followed by the shared DEGs’ basic interpretation including their main biological processes, molecular functions and the signaling pathways they mainly enriched in using Gene ontology analysis (GO) and Kyoto Encyclopedia of Genes and Genomes (KEGG) software [23].

### DEGs PPI network construction and key genes identification

To search the association between different genes, STRING [24], which is short for the Search Tool for the Retrieval of Interacting Genes was used to construct the PPI network among shared DEGs, and the construction criteria was set as confidence score ≥ 0.4 and maximum interactors number = 0.

Followed the DEGs PPI network construction, Molecular Complex Detection (MCODE) plug-in of Cytoscape3.6.0 software [25] was used to analyze the gene function modules based on the network and the analysis cut-off values were set as degree = 2, node score = 0.2, k-core = 2, and max.depth = 100. Using MCODE analysis, we identified the top four gene module (gene clusters sharing similar function) and analyzed the signaling pathways module genes mainly enriched in, meanwhile, the genes that were shared in different modules indicating their probable connecting core genes’ role in the network were highly focused, and the potential core gene’s connectivity degree with surrounding genes was also validated by the Cytohubba plug-in of Cytoscape3.6.0 software.

### Kaplan–Meier survival analysis and clinical pathological features exploration

Kaplan–Meier plotter [26], which contains a total of 54,000 genes in 21 types pan-cancers has been a widely used online service for assessing various genes’ overall survival correlation. In the study, Kaplan–Meier plotter was used to validate the probable core gene’s correlation with ccRCC patients survival and draw the survive curve. Meanwhile, UALCAN [27], which has been an openly accessed online service based on TCGA data was applied to explore gene’s association with ccRCC clinical parameters.

### GEPIA and Oncomine gene expression analysis

GEPIA [28] has been a commonly used online software for worldwide researchers to explore certain genes’ expression and perform survival analysis in various cancers based on the sequencing databases of 9736 cancer and 8587 normal samples from TCGA and GTEx programs. In the study, GEPIA was used to preliminary explore the expression change of FGF1 gene in ccRCC comparing to normal renal tissues.

Besides GEPIA, Oncomine database is also a widely used web-based data mining platform for genes expression analysis. In the study, we additionally used Oncomine to explore the FGF1 expression in broad spectrum human cancers.

### CcRCC tissue microarray production

The ccRCC patients tissues used for microarray production were all collected from local hosptital surgeries at General Surgery Department and sent for pathology examination at our Pathology Department and then stored at Pathology Department Biobank. The Informed consent from the patients as well as the approval by the Hospital Institutional Board were both obtained (Second Hospital of ShanXi Medical University, China).

Further, 104 ccRCC patients samples were picked from the biobank after HE staining confirmation of the disease diagnosis and evaluation of cancer percentage by two local hospital pathologists. Meanwhile, four areas in each sample (including two cancerous and two paracancerous normal areas. Two independent cancerous areas were used to eliminate tumor heterogeneity) were circled under microscope for further study and the receptor wax block were made with 1.5 mm needle according to operating instructions (Chloe, BeiJin, China). Further, tissue microarray was obtained by serial sectioning of the receptor wax block and stored at 4 °C refrigerator (Department of Pathology, Second Hospital of ShanXi Medical University, China).

### Immunohistochemistry (IHC) experiments

#### Regents and tissue samples

IHC experiment was conducted using the ccRCC tissues microarray to validate the gene’s expression difference between cancer and paracancerous normal renal tissues. And it was performed on VENTANA platform (Roche) in local hospital Pathology Department. The primary antibody of FGF1 gene was purchased from abcam (ab179455), and the secondary antibody (Envision /HRP kit) and DAB detection kit were from ZSBG-Bio. Other reagents including H2O2, antigen retrieval citrate solution, phosphate-buffered saline (PBS) and hematoxylin stain were from local hospital Supply Department.

#### IHC experimental protocol

The ccRCC tissue microarray slides were firstly taken out of 4 °C refrigerator and rewarmed at room temperature for 30 min. And then the slides were dewaxed and rehydrated with gradient ethanol followed by antigen retrieval using 10 mmol/l citrate solution. Meanwhile, to inhibit the activity of endogenous peroxidase, the slides were maintained in 0.3% H2O2 containing methanol for 20 min. Further, the slides were soaked in bovine serum albumin for 30 min and then incubated with primary FGF1 antibody (dilution 1:200) overnight at 4 °C followed by a 40 min secondary antibody incubation at 37 °C. Finally, the slides were processed with horseradish peroxidase (HRP) and visualized in DAB for results evaluation.

#### IHC results evaluation

The IHC result was evaluated based on both the microarray tissue cores’ staining intensity and staining area which were scored by two experienced pathologists registered in local hospital Pathology Department with no prior information of the clinical or pathological details of the patients. The staining intensity was scored with the criteria set as: None (0), mild (1), moderate (2) and strong (3), meanwhile, the staining area was classified as: < 5% (0), 6–25% (1), 26–50% (2), 51–75% (3) and > 75% (4). The section’s final score equals the multiplication of staining intensity and staining area, and the final result of each patient’s cancer or paracancerous normal tissue was recorded as the average of two independent microarray cores’ scores, and if the final score < 4, the result was defined as negative, meanwhile, if final score ≥ 4, the result was classified as positive.

### Quantitative real-time PCR (QPCR) experiments

The total mRNA of 30 ccRCC cancer tissues and adjacent paracancerous normal renal tissues (independent of the 104 cases used for microarray production) were extracted using RNAiso-Plus (TAKARA, DaLian, China). And then1 μg extracted mRNA was used for cDNA synthesis with cDNA synthesis kit (TAKARA, DaLian, China) according to operating instruction. Further, qPCR was performed on Roche z 480 and the primers used were listed as below:

FGF1:

Former: CACATTCAGCTGCAGCTCAG

Reverse: TGCTTTCTGGCCATAGTGAGTC

GAPDH:

Former: AGAAGGCTGGGGCTCATTTG

Reverse: AGGGGCCATCCACAGTCTTC

The PCR cycling condition was set as: 95 °C 5 min for 1 cycle; 95 °C 5 s, 62 °C 30 s, and 72 °C 34 s for 35 cycles followed by the melting curve stage. And the relative gene expression in each sample was recorded as the average 2^ − ΔΔCT calculation result of three replicates. Further, T-test was used for detailed statistical analysis. P < 0.05 was considered statistically significant.

### Gene’s physicochemical properties

ProtParam [29] is a newly developed online software and it is commonly used for computing the physical and chemical parameters of certain proteins including their molecular weights, theoretical isoelectric point, aminoacid composition, extinction coefficient, estimated protein half life, protein instability index and grand average of hydrophilicity.

Besides ProtParam, ProtScale [30] is also a widely used online service for computing the aminoacid scales on a selected protein, and the most frequently used scales are the hydrophobicity, hydrophilicity and secondary structure conformational parameters.

In addition to ProtParam and ProtScale, Human Protein Atlas [31] is also an openly accessed online service for targeting proteins information. Using integration of various technologies, including antibody-based imaging, mass spectrometry-based proteomics, transcriptomics and systems biology, Human Protein Atlas is aiming to map various human proteins in cells, tissues and human organs.

In the study, we used Human Protein Atlas to preliminary explore the cellular location of FGF1 protein in ccRCC cells. And ProtParam and ProtScale were used to interpret the gene’s basic physicochemical parameters.

### Related signaling pathways and potential biological functions analysis

Gene ontology analysis (GO) and Kyoto Encyclopedia of Genes and Genomes (KEGG) have been two effectively used online services for annotating lists of genes and interpreting networks of signaling pathways. In the study, to explore the potential biological functions and probable signaling pathways of FGF1 gene, STRING was firstly used to reveal the surrounding genes that relate mostly with FGF1. And then, GO and KEGG were used to annotate the the signaling pathways that centered on FGF1, and the gene’s potential biological functions were also preliminary explored.

## Results

### Identification of 192 DEGs in ccRCC comparing to normal renal tissues

**Fig. 1:**
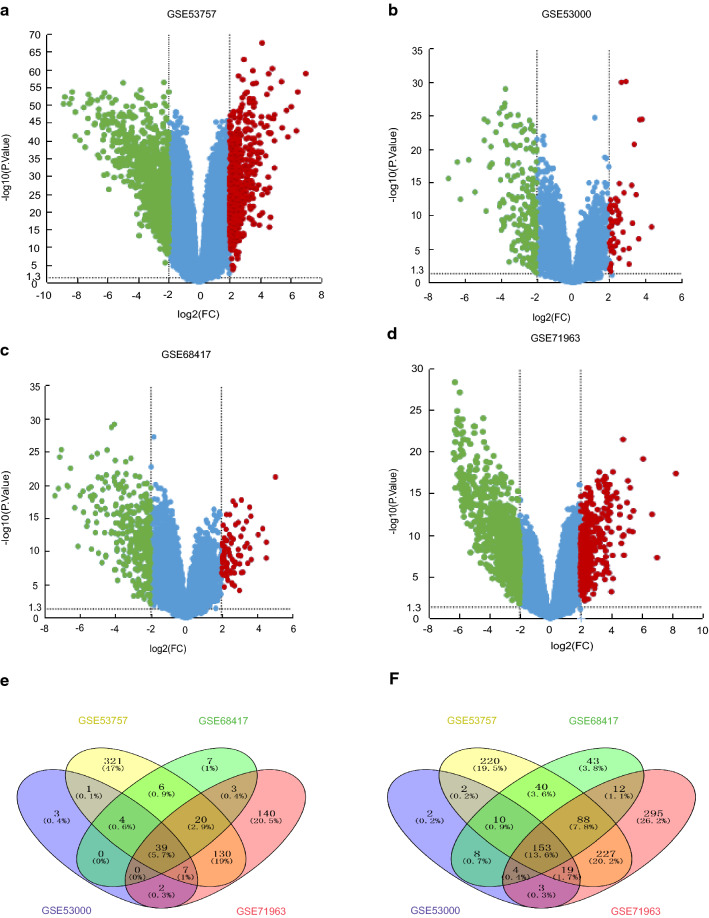
The DEGs screened from GEO expression profiles. Up-regulated (red-colored spots) and down-regulated (green-colored spots) DEGS in ccRCC comparing to normal renal tissues were screened from GEO profiles **a** GSE53000, **b** GSE53757, **c** GSE68417 and **d** GSE71963 respectively. **e** 39 up-regulated and **f** 153 down-regulated DEGs were shared in all four GEO expression profiles

Four cDNA expression profiles from GEO database were used to screen the DEGs in ccRCC vs. normal renal tissues, and eventually a total of 1286 (Fig. 1a), 1142 (Fig. 1b), 437 (Fig. 1c) and 257 (Fig. 1d) DEGs were identified in GSE53757, GSE71963, GSE68417 and GSE53000 profiles respectively. And of all the DEGs, 192 genes were shared in all four profiles including 39 genes that were shown to be up-regulated (Fig. 1e) and 153 down-regulated genes (Fig. 1f) in cancer comparing to normal tissues (Additional file 1: Table S2).

### Basic interpretation of 192 DEGs by GO and KEGG

**Fig. 2:**
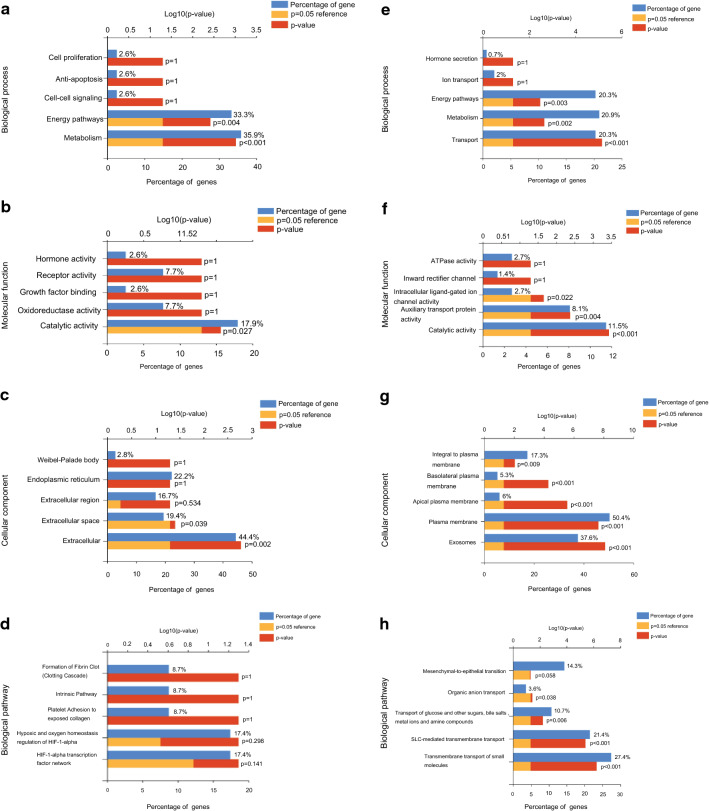
GO and KEGG analysis of DEGs in ccRCC. **a** The biological processes, **b** molecular functions, **c** cellular components and **d** biological pathways the up-regulated DEGS were mainly enriched in. **e** The biological processes, **f** molecular functions, **g** cellular components and **h** biological pathways the down-regulated DEGS were mostly focused on

To preliminary explore the biological functions of the 192 DEGs, GO and KEGG analysis were performed. Excitingly, GO results showed that the biological processes that both the 39 up-regulated (Fig. 2a) and 153 down-regulated genes (Fig. 2e) mainly enriched in were metabolism and energy regulation pathways. Meanwhile, the cellular component of 39 up-regulated were mostly focused on extra cellular (Fig. 2c), and the molecular functions were primary oxidoreductase and receptor activities related (Fig. 2b). Further, KEGG/biological pathway analysis showed the up-regulated DEGs were mostly enriched in HIF-1α related hypoxia and oxygen homeostasis regulating signaling pathways (Fig. 2d).

And as for the 153 down-regulated genes, the cellular component were mainly enriched in plasma membrane (Fig. 2g), the molecular function were mostly focused on catalytic and auxiliary transport protein activities (Fig. 2f), and the KEGG signaling were mainly trans-membrane transport of small molecules, for instance glucose, bile salts and organic related (Fig. 2h).

### FGF1 gene works as a core gene in DEGs PPI network

**Fig. 3:**
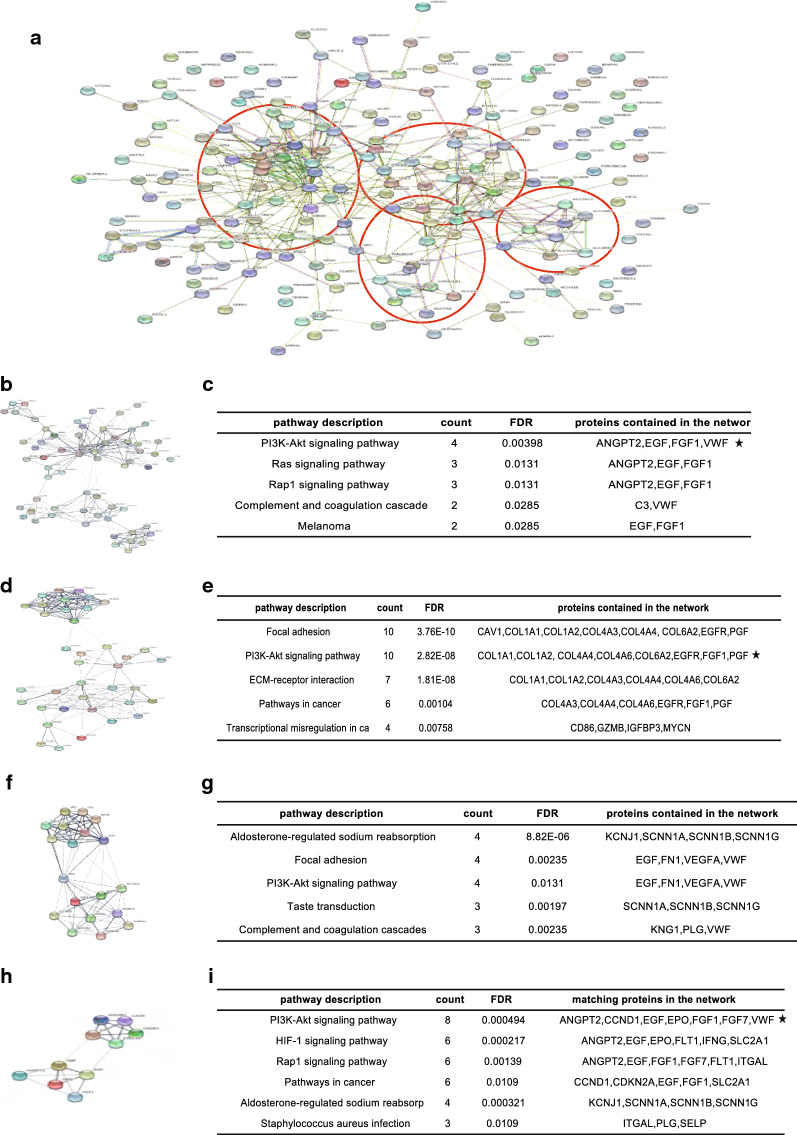
Genes’ function modules analysis based on DEGs’ PPI network. **a** The PPI network of 192 DEGs and four main function modules analyzed based on the network (four red circles and each represents one gene module). **b**, **d**, **f**, **h** The diagrammatic sketch and **c**, **e**, **g**, **i** containing main signaling pathways as well as involving genes of four main modules in the PPI network. (* The FGF1 gene involved PI3K-AKT signaling pathway was revealed in 3/4 modules.)

To further scale down the “candidate” genes and identify the potential key genes regulating ccRCC development, we construct the PPI network of 192 shared DEGs for further function modules analysis, thus understanding the interaction between different genes (Fig. 3a). And based on the PPI network, four modules were identified revealing signaling pathways that DEGs were mainly enriched in, interestingly, an FGF1 gene involving PI3K-AKT signaling was identified in 3/4 modules suggesting it’s potential “core” position in the network (Fig. 3b–i).

**Fig. 4:**
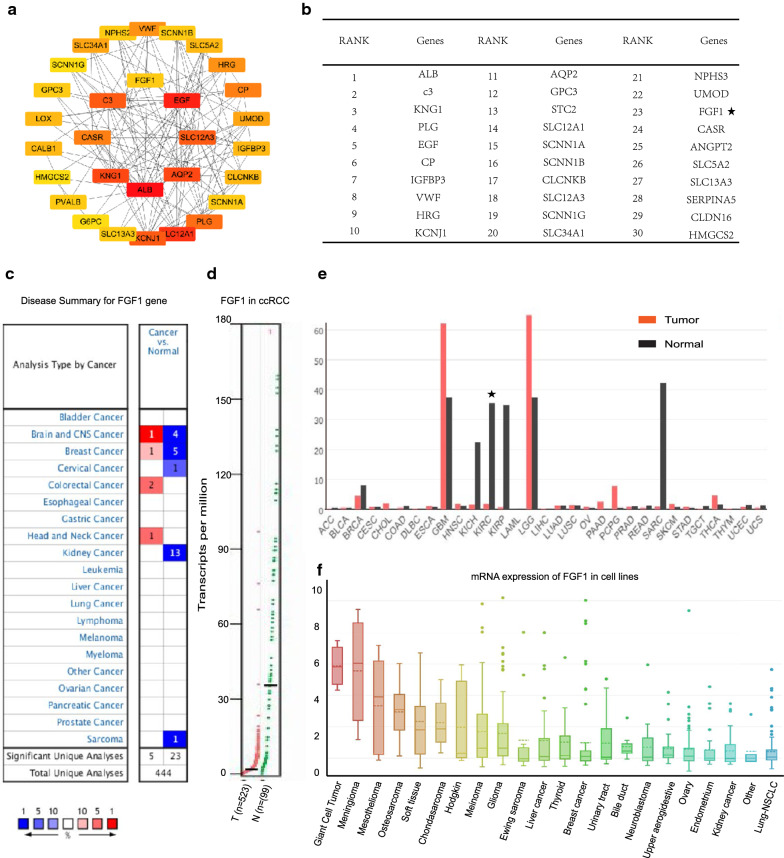
Aberrant FGF1 loss of expression in ccRCC comparing to normal renal tissues. **a** Top 30 genes in the PPI network with high connectivity with surrounding genes (higher color represents stronger connectivity). **b** The top 30 genes in the PPI network with high connectivity with surrounding genes listed in descending order. (* FGF1 gene is 23rd of the 30 top genes). **c** Expression of FGF1 in different types of human cancers revealed by Oncomine analysis. Different colored squares indicated the numbers of datasets with FGF1 mRNA over-expressed (red) or down-expressed (blue) in cancer vs. normal tissue. **d** Aberrant loss of expression of FGF1 in ccRCC comparing to normal renal tissues revealed by GEPIA analysis. **e** Expression of FGF1 in different types of human cancers by GEPIA analysis (*FGF1 expression in KIRC which is short kidney renal clear cell carcinoma, another name of ccRCC). **f** Expression of FGF1 in different cancer cell lines

Additionally, to validate the “core” position of FGF1 gene in the network, the connectivity between different genes in the PPI were also explored, and the result supported FGF1 as one of the top 30 genes with high connectivity with surrounding genes (Fig. 4a, b). Moreover, Oncomine analysis revealed that although FGF1 expression various in different human cancers, multiple previous studies supported the FGF1 loss of expression in kidney cancers (Fig. 4c, d). And another analysis performed by GEPIA also showed consistent results that FGF1 expression various in different human tumors, for instance, the expression was higher glioblastoma and brain lower grade glioma comparing to paired normal tissues, but its expression in other tumors including ccRCC is aberrant lost (Fig. 4e).

Additionally, not only in the solid tissues, FGF1 expression was significantly lower in ccRCC cell lines comparing to other cancer cells (Fig. 4f).

Both GEPIA and Oncomine results supported the aberrant loss of expression of FGF1 in ccRCC comparing to normal kidney in both solid tissues and cell lines.

### Aberrant FGF1 loss of expression in ccRCC

**Fig. 5:**
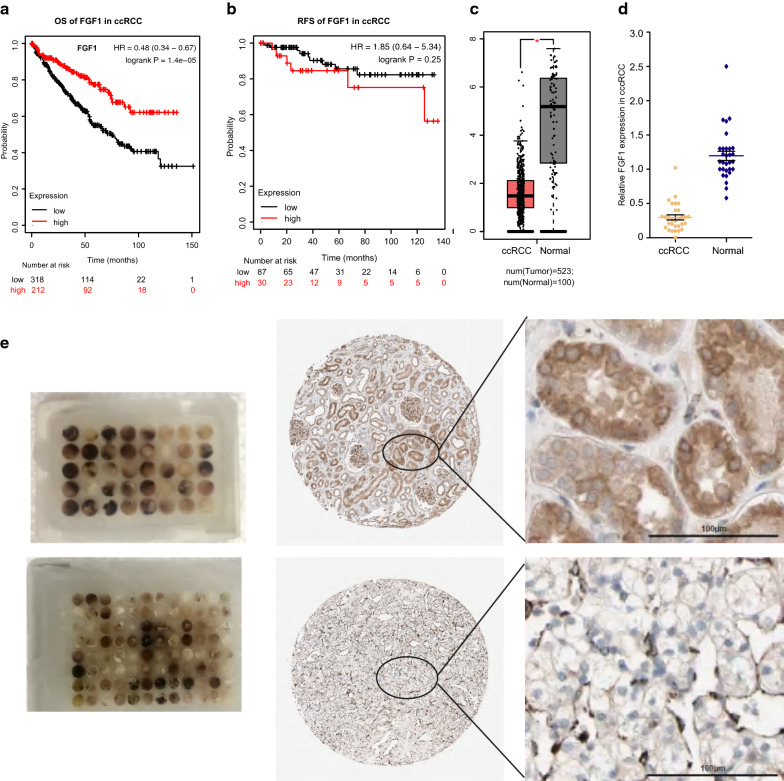
Expression level of FGF1 in ccRCC verses normal kidney tissues revealed by local hospital samples experiments. **a** Overall survival analysis of FGF1 in ccRCC by Kaplan–Meier survival analysis. **b** Recurrence free survival analysis of FGF1 in ccRCC by Kaplan–Meier survival analysis. **c** FGF1 expression in ccRCC comparing to normal kidney tissues revealed by GEPIA analysis. **d** FGF1 expression in ccRCC comparing to normal kidney tissues revealed by QPCR experiment using 30 cases of local hospital patients samples. **f** 104 Local hospitalized ccRCC cancer samples were made into tissue arrays (as the left line graphics). The relative expression of FGF1 is qualified in ccRCC (the upper two graphics in the right) comparing to normal renal tissues (the lower two graphics in the right) by IHC experiment using ccRCC tissue microarrays

To reveal the clinical value of FGF1 loss of expression in ccRCC. Kaplan–Meier plotter survival analysis was firstly conducted. And the overall survival analysis based on 530 kidney renal clear cell carcinoma samples revealed that FGF1 statistical significantly correlates with patients overall survival (OS), but not recurrence free survival (RFS), higher FGF1 gene expression directly associated with better patients overall survival indicating its potential tumor inhibitor function in ccRCC (Fig. 5a, b).

To reveal the expression of FGF1 in ccRCC, besides the previous online analysis, IHC as well as QPCR experiments using local hospital patients tissues were also conducted (Detailed samples information see Additional file 2: Table S4). Consistent with the GEPIA online analysis (Fig. 5c), the result of qRT-PCR which was conducted using 30 local hospital ccRCC and paired normal renal tissues also supported the FGF1 loss of expression in cancer (Fig. 5d).

Meanwhile, the immunohistochemistry (IHC) carried out in 104 local hospital ccRCC and paired normal renal tissues (different from the 30 samples used in qRT-PCR experiment) also revealed that FGF1 expression was significantly lower in cancer comparing to normal tissues. Significant loss of expression (less than 1%) was observed in ccRCC versus the much higher expression ratio (48.7%) in normal tissues (P < 0.01) (Fig. 5e).

### The association between FGF1 gene and ccRCC clinical features

**Fig. 6:**
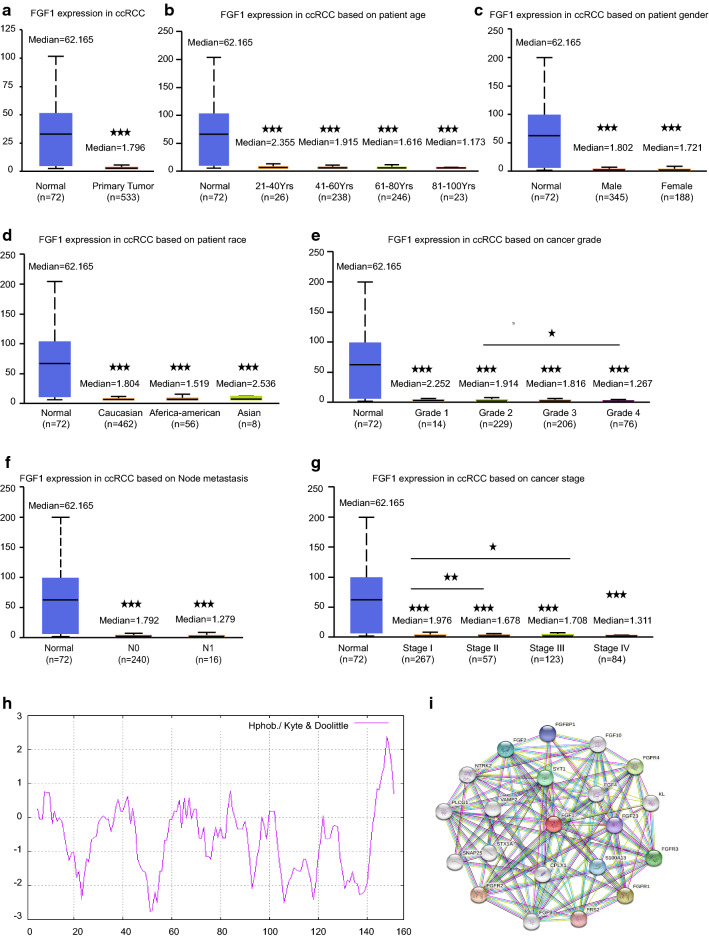
The association between FGF1 expression and ccRCC clinical parameters. **a** Relative FGF1 expression in ccRCC versus normal renal tissues. And the association between FGF1 expression and ccRCC **b** patients age, **c** gender, **d** race, **e** tumor grade, **f** lymph node metastasis and **g** tumor stage. (*p < 0.05, **p < 0.01, ***p < 0.001. The first layer * which is right above the error bar representing comparison to normal group, and the above layers * which were above a secondary line represent the comparison between corresponding groups that were covered by the line). **h** The hydrophilcity/hydrophobicity analysis of FGF1 protein. **i** FGF1 centered PPI network representing the genes that were mostly related to FGF1

The association between FGF1 expression and ccRCC clinicopathological parameters was analyzed using Ualcan, which is an public online service based on TCGA data containing a whole of 533 ccRCC and 75 normal renal samples. The analysis result showed not only that FGF1 expresses much less in cancer comparing to normal renal tissues (Fig. 6a), but also the expression tends to decrease as the cancer grade and stage advancing although the difference was not statistical significant. Also, the expression of FGF1 tends to be lost more in older patients with lympho nodes metastasis than patients with younger age and no lympho metastasis, but the difference was not statistical significant. Meanwhile, no significance relationship was found between FGF1 expression and patients race and gender (Fig. 6b–g).

Besides Ualcan online analysis, we also downloaded the original patients data from TCGA website(containing 539 ccRCC and 72 normal renal samples, detailed TCGA patients barcods in Additional file 1: Table S3) to validate the clinical parameters of FGF1 in ccRCC. Another interesting fact was found that FGF1 expression decreased in patients after radiation therapy, which was consistent with the blood test which showed that white cell count was much higher in patients with high FGF1 expression (Table 1).

**Table 1:** The association between FGF1 and ccRCC clinical pathological features

Parameters	FGF1 (%)		P value
	−	+	
Gender			
Female	69 (39.9)	104 (60.1)	0.608
Male	122 (37.5)	203 (62.5)	
Race			
White	157 (37.0)	267 (63.0)	0.154
Black	30 (50.0)	30 (50.0)	
Asia	3 (37.5)	5 (62.5)	
Chemotherapy			
Yes	185 (39.0)	289 (61.0)	0.168
No	6 (25.0)	18 (75.0)	
White cell count			
Low	5 (62.5)	3 (37.5)	0.028
Normal	96 (39.5)	147 (60.5)	
Elevated	48 (29.1)	117 (70.9)	
Radiation			
No	165 (36.3)	290 (63.7)	0.002
Yes	26 (60.5)	17 (39.5)	
Tumor grade			
I	99 (33.3)	198 (66.7)	0.042
II	64 (32.0)	136 (68.0)	
III	50 (43.1)	66 (56.9)	
IV	50 (45.5)	60 (54.5)	
T stage			
T1	91 (33.2)	183 (66.8)	0.065
T2	24 (41.4)	34 (58.6)	
T3	71 (45.5)	85 (54.5)	
T4	5 (50.0)	5 (50.0)	
N stage			
N0	84 (38.4)	135 (61.6)	0.123
N1	9 (64.3)	5 (35.7)	
M stage			
M0	143 (35.0)	265 (65.0)	0.005
M1	31 (52.5)	28 (47.5)	

### Physicochemical properties of FGF1 gene

Two online services ProtParam and ProtScale were used to predict FGF1′s physicochemical properties, and the results revealed that FGF1 protein is composed of 155 amino acids, including 19 negatively charged amino acid residues (ASP+Glu) and 18 positively charged amino acid residues (Arg+Lys). The molecular formula of FGF1 protein is C777H1208N210O238S5, the molecular weight is 17.5KD, and the theoretical isoelectric point is 6.51.

Meanwhile, the estimated half-life of FGF1 protein is 30 h in mammals and the instability index is computed to be 40.67 indicating the protein tends to be cellular unstable.

Additionally, ProtParam computed the hydrophobic value of FGF1 is 73.61 and the average hydrophilicity is − 0.620. ProtScale also revealed that FGF1 protein harbors several hydrophilic regions and shall be classified as a hydrophilic protein (Fig. 6h). Also, the result of Protein Atlas analysis supported FGF1 locating both in the nucleoplasm and is predicted to be secreted, suggesting its potential biological function as a hydrophilic signaling pathway particle.

### FGF1 gene centered biological functions and related signaling pathways

To further explore the potential biological functions of FGF1 gene in ccRCC and the probable signaling pathways involved, GO and KEGG analysis were performed. And GO results showed that the biological processes FGF1 gene participated in were mainly focused on fibroblast growth factor receptor activities, phosphatidylinositol-3-phosphate biosynthetic processes and phosphatidylinositol-3-phosphate biosynthetic associated processes. And the molecular functions FGF1 played were most enriched in fibroblast growth factor receptor binding, 1-phosphatidylinositol-3-kinase activity, phosphatidylinositol-4,5-bisphosphate 3-kinase activity and protein tyrosine kinase activities (Fig. 6i, Table 2).

**Table 2:** Biological Process events centered on FGF1

Description	Counts	Background gene counts	FDR	Matching proteins in the network
1-Phosphatidylinositol-3-kinase activity	18	44	2.42e−36	FGF4, FGF20, FGF23, FGF2, FGF10, FGF7, FGFR4, FGF19, FGF8, FGF3, FGFR3, KL, FGF9, GRB2, FGFR1, FGFR2, FRS2, FGF1
Phosphatidylinositol-4,5- bisphosphate 3-kinase activity	18	68	6.57e−34	FGF4, FGF20, FGF23, FGF2, FGF10, FGF7, FGFR4, FGF19,FGF8, FGF3, FGFR3, KL, FGF9, GRB2, FGFR1, FGFR2, FRS2, FGF1
Ras guanyl-nucleotide exchange factor activity	18	243	3.91e−25	FGF4, FGF20, FGF23, FGF2, FGF10, FGF7, FGFR4, FGF19, FGF8, FGF3, FGFR3, KL, FGF9, GRB2, FGFR1, FGFR2, FRS2, FGF1
Protein tyrosine kinase activity	17	180	3.34e−25	FGF4 ,FGF20 ,FGF23, FGF2, FGF10, FGF7, NTRK2, FGFR4, FGF8, FGF3, FGFR3, FGF9, GRB2, FGFR1, FGFR2, NTRK1, FGF1
Growth factor receptor binding	14	131	3.40e−21	FGF4, FGF20, FGF23, FGF2, FGF10, FGF7, FGF19, FGF8, FGF3, KL, FGF9, GRB2, FRS2, FGF1
Fibroblast growth factor receptor binding	13	27	7.40e−27	FGF4, FGF20, FGF23, FGF2, FGF10, FGF7, FGF19, FGF8, FGF3, KL, FGF9, FRS2, FGF1
Transmembrane receptor protein tyrosine kinase activity	6	61	4.88e−09	NTRK2, FGFR4, FGFR3, FGFR1, FGFR2, NTRK1

Meanwhile, KEGG analysis revealed the signaling pathways FGF1 gene involved were mainly RAS signaling, Rap1 signaling, PI3K-AKT signaling and MAPK signaling pathways related (Table 3). Considering our previous gene module analysis based on PPI network which showed that FGF1 involved PI3K-AKT signaling shall play a core role in the network, it’s of potential clinical value to further investigate the potential drug-targeting role of FGF1 gene or other FGF1 interacted PI3K-AKT signaling proteins in the development of ccRCC, thus aiding more precise understanding of the disease.

**Table 3:** KEGG signaling pathways centered on FGF1

Term description	Counts	Background gene counts	FDR	Matching proteins in the network
Ras signaling pathway	19	228	4.33e−27	FGF4, FGF20, FGF23, PLCG1, FGF2, FGF10, FGF7, NTRK2, FGFR4, FGF19, FGF8, FGF3, FGFR3, FGF9, GRB2, FGFR1, FGFR2, NTRK1, FGF1
MAPK signaling pathway	18	293	1.85e−23	FGF4, FGF20, FGF23, FGF2, FGF10, FGF7, NTRK2, FGFR4, FGF19, FGF8, FGF3, FGFR3, FGF9, GRB2, FGFR1, FGFR2, NTRK1, FGF1
PI3K-AKT signaling pathway	18	348	2.42e−22	FGF4, FGF20, FGF23, FGF2, FGF10, FGF7, NTRK2, FGFR4, FGF19, FGF8, FGF3, FGFR3, FGF9, GRB2, FGFR1, FGFR2, NTRK1, FGF1
Regulation of actin cytoskeleton	15	205	1.56e−20	FGF4, FGF20, FGF23, FGF2, FGF10, FGF7, FGFR4, FGF19, FGF8, FGF3, FGFR3, FGF9, FGFR1, FGFR2, FGF1
Signaling pathways regulating pluripotency of stem cells	6	138	7.10e−7	FGF2, FGFR4, FGFR3, GRB2, FGFR1, FGFR2
EGFR tyrosine kinase inhibitor resistance	5	78	1.36e−6	PLCG1, FGF2, FGFR3, GRB2, FGFR2
Insulin secretion	3	84	0.0019	STX1A, SNAP25, VAMP2

## Discussion

Renal cell carcinoma has been a common malignant tumor of urinary tract, ranking only second to bladder carcinoma in morbidity of all adults urinary tract malignant tumors. Although the clinical treatment situation is still challenging given the tumor heterogeneity and evolutionary nature of cancer [32, 33], the increasing developing molecular pathology has been bringing promising effect for ccRCC in both molecular diagnosis and targeting treatment. Especially in current precise medicine era, the various bioinformatic analysis tools has been making it more practicable for worldwide researchers to explore the molecular genetic abnormalities in cancers [34, 35]. In the study, we combine used four different GEO cDNA expression profiles together with multiple bioinformatic analysis methods to explore the potential new prognostic indicators in ccRCC development, and we identified a specific FGF1 gene which was proved to be aberrant lost expression in ccRCC comparing to normal renal tissues, and the FGF1 lose of expression was indicated to be a worse overall survival indicator in ccRCC patients.

GEO database has been one of the most commonly used public databases for worldwide researchers to explore the genetic abnormalities in various cancers [36–39]. In the study, we firstly picked four different cDNA expression profiles GSE53757, GSE53000, GSE71963 and GSE68417 from GEO database to analyze the differently expressed genes in ccRCC comparing to normal renal tissue, and the result revealed 192 genes that were shared in four profiles including 39 up-regulated and 153 down-regulated genes. Interestingly, although mainly enriched in different cellular locations and involved in various signaling pathways, both the up and down regulated DEGs were mostly participated in metabolism and energy regulation related biological processes.

The mainly focus of DEGs on the metabolism related biological processes in the study supported the importance of the elaborate network of energy consuming in cancer development. Metabolomics has been a classic theory in cancer research based on the well known fact that even in the presence of oxygen, cancer cells perform less energy-efficient glycolysis process termed as aerobic glycolysis or Warburg effect [40, 41]. Although the detailed reasons for Warburg effect are still unclear, one of the theories is that increased glycolysis may provide cancer cells easier access to accumulation of essential metabolic precursors they need for rapid cell proliferation [42–44].

To further scale down the “candidate” responsible genes and identify the potential “key” gene in ccRCC development, the PPI network of 192 DEGs was constructed to visualize the relationship between genes, and then gene function module analysis was successively performed. As a result, four gene modules involving various signaling pathways were identified based on the PPI network, excitingly, a FGF1 gene involving PI3K-Akt signaling pathway was shared in 3/4 modules indicating its potential core position in the network.

What’s more, the connectivity degree analysis between DEGs with surrounding genes also supported FGF1 gene as one of the top 30 genes with highest connectivity with other DEGs in the network. FGF1 gene, which is short for fibroblast growth factor 1, is one of the members of fibroblast growth factor (FGF) family and it has been reported to play important roles in the regulation of cell survival, cell division, angiogenesis, cell differentiation and migration [45]. In the study, the potential function of FGF1 gene in ccRCC development was explored.

Firstly, Kaplan–Meier survival analysis based on TCGA data revealed that FGF1 gene expression statistical significantly correlates with ccRCC patients overall survival, higher FGF1 expression was associated with better survival, suggesting its potential tumor suppressor function.

Then, to explore the expression of FGF1 in ccRCC comparing to normal renal tissues, both online database analysis and experiments based on local hospital samples were conducted. Both online GEPIA and Oncomine analysis indicated that although FGF1 expression various in different cancers, it was aberrant lost in ccRCC. Meanwhile, our IHC experiments conducted on tissue microarray which was produced using 104 local patients samples supported the loss of expression ratio (less than 1%) in ccRCC comparing to normal renal tissues (48.7%). What’s more, QPCR experiment performed using 30 different patients samples also validated that FGF1 expressed less in cancer comparing to matched normal tissues.

Since the sample number being used for our IHC and QPCR experiments was relatively low (104 cases for IHC experiment and 30 for QPCR experiment), and the patients with greater than 2, 3, 4 and 5 years follow-up was 70, 34, 17 and 13 respectively, the medial follow-up of the 134 patients was 33 months. To avoid the limitations of relatively small number samples and short duration of follow-up, an online service UALCAN which is based on TCGA data containing a total of 533 primary ccRCC and 72 normal renal samples was used for further analyzing the association between FGF1 and ccRCC clinical parameters. And the result showed that FGF1 loss expression in broad-spectrum ccRCC patients despite of the race, age, cancer grade and stage, and no significance relationship was found between FGF1 expression and patients gender.

Further, to explore the potential biological function of FGF1 in ccRCC development, we computed the basic physicochemical parameters of the protein, which result revealed that FGF1 is a hydrophilic protein weighting 17.5KD, and the protein mainly locates in the nucleoplasm or to be secreted out of cells, the estimated half-time is 30 h and tend to be unstable.

Meanwhile, the biological processes FGF1 gene participated in were mainly focused on fibroblast growth factor receptor activities and phosphatidylinositol-3-phosphate biosynthetic related processes, and the FGF1 centered signaling pathways were mostly RAS signaling, PI3K-AKT signaling and Rap1 signaling pathways. Given the result of our function module analysis which indicated that FGF1 gene involved PI3K-AKT signaling shall be in the core position of the DEGs PPI network, it’s of potential clinical value to further investigate the detailed function and the mechanism behind FGF1 related PI3K-AKT signaling pathways in the regulation of ccRCC development.

Actually, PI3K-Akt signaling has been commonly known to regulate insulin-based glucose metabolism and mutations of the pathway genes resulting in aberrant signaling activation, thus leading to higher amount of glucose uptake [46]. And activation of PI3K-Akt signaling provokes the expression of HIF-1α, which is a transcription factor generally known be involved in the cellular adaption to hypoxia and modulates cellular anaerobic metabolism [47].

Moreover, FGF1 expression was reported to be inhibited in diabetic nephropathy, and exogenous recombinant FGF1 protein not only has excellent function of reducing blood glucose level in type 2 diabetes mellitus, but also has a very obvious improvement effect on recovering the impaired diabetic renal function [48]. Interestingly, although FGF1 has no hypoglycemic effect on type 1 diabetes mellitus, it can also improve the renal function of type 1 diabetes mellitus indicating the improvement function of FGF1 on diabetic nephropathy exists independently of the hypoglycemic effect [49]. What’s intriguing is that there’s currently no evidence of association between ccRCC and diabetic nephropathy, sharing a similar genetic abnormality (loss of FGF1 expression) might provoke worldwide renal disease researchers’ interest for further analysis.

However, although above results shall provide meaningful insights into better understanding of ccRCC, it’s not yet enough to classify FGF1 or other PI3K-AKT signaling proteins as new potential drug targets in ccRCC. To distinguish gene aberrations that can cause the disease and may serve as drug targets with those being closely linked to the disease and consequently are associated with the disease development, further comprehensive experiments and clinical trials are needed to be performed.

## Conclusion

In conclusion, based on GEO database, we analyzed 192 DEGs in ccRCC comparing to normal renal tissues, and FGF1 gene and PI3K-AKT signaling was identified as a core signaling in DEGs’ PPI network. Both online public data analysis and local hospital IHC as well as QPCR experiments validated the aberrant loss expression of FGF1 in ccRCC comparing to normal tissues. Kaplan–Meier overall survival analysis revealed that low FGF1 expression was associated with worse patients survival. Additionally, FGF1 centered biological processes and signaling pathways were preliminary explored. Comprehensive studies and clinical trials are needed to confirm the findings before promoting the clinical utility of FGF1as a new drug target and prognosis indicator in ccRCC.

## Supplementary Information

**Additional file 1:Table S1.** Detailed GEO datasets information used for DEGs analysis. **Table S2.** GEO data revealed 192 DEGs in ccRCC comparing to normal renal tissues. **Table S3.** The TCGA patients barcode for 611 ccRCC samples.

**Additional file 2: Table S4.** Detailed patients information used for IHC and QPCR experiments.

## Data Availability

All data generated or analyzed during this study are included in this published article.

## Abbreviations

RCC: Renal cell carcinoma
ccRCC: Clear cell renal cell carcinoma
GEO: Gene Expression Omnibus
DEGs: Differently expressed genes
IHC: Immunochemistry experiment
QPCR: Quantitative real time polymerase chain reaction
NGS: Next generation sequencing
GO: Gene ontology
KEGG: Kyoto Encyclopedia of Gene and Genome
PPI: Protein–protein interaction
OS: Overall survival rate
STRING: Search Tool for the Retrieval of interacting Genes
MCODE: Molecular Complex Detection
FGF1: Fibroblast growth factor 1
