# Supplementary material for: Bioinformatic analysis identifying FGF1 gene as a new prognostic indicator in clear cell Renal Cell Carcinoma
Source: Cancer Cell Int. 2021 Apr 17;21:222. doi: 10.1186/s12935-021-01917-9 (PMC8052755; doi:10.1186/s12935-021-01917-9)
Supplement: Supplementary file 1 — Additional file 1:Table S1. Detailed GEO datasets information used for DEGs analysis. Table S2. GEO data revealed 192 DEGs in ccRCC comparing to normal renal tissues. Table S3. The TCGA patients barcode for 611 ccRCC samples. [file 12935_2021_1917_MOESM1_ESM.docx]

**Supplementary Table 1. Detailed GEO datasets information used for DEGs analysis**

| **GEO Dataset** | **Public data** | **Connector** | **Sample amount** | **Accessing website** |
| --- | --- | --- | --- | --- |
| GSE53000 | Jan 06, 2014 | Pierre Martinez | 56 ccRCC T  6 kidney N | https://www.ncbi.nlm.nih.gov/geo/query/acc.cgi?acc=GSE53000 |
| GSE68417 | Jan 04, 2016 | Bryan Thibodeau | 29 ccRCC T  14 kidney N | https://www.ncbi.nlm.nih.gov/geo/query/acc.cgi?acc=GSE68417 |
| GSE53757 | Jan 03, 2014 | John A Copland | 72 ccRCC T  72 kidney N | https://www.ncbi.nlm.nih.gov/geo/query/acc.cgi?acc=GSE53757 |
| GSE71963 | Aug 09, 2016 | Mika Takahashi | 32 ccRCC T  16 kidney N | https://www.ncbi.nlm.nih.gov/geo/query/acc.cgi?acc=GSE71963 |

**Supplementary Table 2 GEO data revealed 192 DEGs in ccRCC comparing to normal renal tissues**

| **List of DEGs in ccRCC versus normal renal tissues** | | | | | |
| --- | --- | --- | --- | --- | --- |
| **Up**  **regulated** | ANGPT2 | DIRAS2 | HK2 | PCSK6 | ST8SIA4 |
|  | ANGPTL4 | DNAH11 | IDO1 | PLA2G7 | STC2 |
|  | APOC1 | EGLN3 | IGFBP3 | QRFPR | TMEM45A |
|  | C3 | ENO2 | LAMA4 | SCARB1 | TNFAIP6 |
|  | CA9 | ENPP3 | LOX | SCD | VWF |
|  | CD36 | ESM1 | LOXL2 | NETO2 | SEMA5B |
|  | CP | FABP7 | NDUFA4L2 | GAS2L3 | SLC6A3 |
|  | AHNAK2 | CYP2J2 | HILPDA | NNMT |  |
| **Down**  **regulated** | ACPP | ALDOB | CDH9 | CWH43 | DPEP1 |
|  | ACSF2 | AP1M2 | CHL1 | CYP17A1 | EGF |
|  | ADGRF1 | AQP2 | CLCNKB | CYP24A1 | EHF |
|  | ADH1B | ATP6V0A4 | CLDN16 | CYP2B6 | ENPP6 |
|  | ADH1C | ATP6V0D2 | CLDN8 | CYP4A11 | EPCAM |
|  | ADH6 | ATP6V1B1 | CLIC5 | CYP4F2 | ERBB4 |
|  | AFM | ATP6V1C2 | CNTN1 | DAO | ERP27 |
|  | AIF1L | C7 | CNTN3 | DCN | ESRRG |
|  | ALB | CALB1 | CRHBP | DEFB1 | F11 |
|  | ABAT | ALDH6A1 | CASR | CTXN3 | DIO1 |
|  | FAM3B | GPAT3 | HSD11B2 | MFSD4A | PCP4 |
|  | FAM83B | GPC3 | HSPA2 | MIOX | PIGR |
|  | FGF1 | GRB14 | IYD | MPPED2 | PIK3C2G |
|  | FGF9 | GRHL2 | KCNJ1 | MT1G | PLA2R1 |
|  | FREM1 | GSTM3 | KCNJ10 | MT1H | PLCL1 |
|  | FXYD4 | HAO2 | KCNJ15 | MUC15 | PLG |
|  | G6PC | HEPACAM2 | KNG1 | NPHS2 | PLPP4 |
|  | GATA3 | HMGCS2 | KRT7 | OGDHL | PLPPR1 |
|  | GGT6 | HPD | LIPH | PAPPA | PRLR |
|  | FAM151A | GMPR | HRG | MAL | PCK1 |
|  | PTH1R | SCNN1B | SLC22A6 | SLC7A13 | TMEM174 |
|  | PVALB | SCNN1G | SLC22A7 | SLC7A8 | TMEM178A |
|  | RAB25 | SERPINA5 | SLC22A8 | SOSTDC1 | TMEM30B |
|  | RALYL | SFRP1 | SLC26A7 | SPTBN2 | TMEM45B |
|  | RANBP3L | SHISA3 | SLC2A12 | STAP1 | TMEM52B |
|  | RHCG | SLC12A1 | SLC34A1 | SUCNR1 | TMPRSS4 |
|  | RNF150 | SLC12A3 | SLC44A4 | TACSTD2 | TSPAN8 |
|  | RPS6KA6 | SLC13A3 | SLC47A2 | TCF21 | UMOD |
|  | SCN2A | SLC15A2 | SLC4A1 | TFAP2B | UPP2 |
|  | PSAT1 | SCNN1A | SLC22A13 | SLC5A2 | TFCR2L1 |
|  | VTCN1 | WDR72 | XPNPEP2 |  |  |

**Supplementary Table 3. The TCGA patients barcode for 611 ccRCC samples**

| TCGA-A3-3387 | TCGA-B8-4620 | TCGA-CW-5591 | TCGA-CZ-5467 | TCGA-A3-3317 | TCGA-A3-3359 |
| --- | --- | --- | --- | --- | --- |
| TCGA-B0-4700 | TCGA-B8-4622 | TCGA-CW-6087 | TCGA-CZ-5468 | TCGA-A3-3319 | TCGA-A3-3362 |
| TCGA-B0-4712 | TCGA-B8-5549 | TCGA-CW-6088 | TCGA-CZ-5469 | TCGA-A3-3320 | TCGA-A3-3363 |
| TCGA-B0-5402 | TCGA-B8-5552 | TCGA-CW-6090 | TCGA-CZ-5470 | TCGA-A3-3322 | TCGA-A3-3365 |
| TCGA-B0-5690 | TCGA-CJ-5672 | TCGA-CZ-4863 | TCGA-CZ-5982 | TCGA-A3-3323 | TCGA-A3-3367 |
| TCGA-B0-5691 | TCGA-CJ-5676 | TCGA-CZ-4864 | TCGA-CZ-5984 | TCGA-A3-3324 | TCGA-A3-3370 |
| TCGA-B0-5694 | TCGA-CJ-5677 | TCGA-CZ-4865 | TCGA-CZ-5985 | TCGA-A3-3325 | TCGA-A3-3372 |
| TCGA-B0-5696 | TCGA-CJ-5678 | TCGA-CZ-5451 | TCGA-CZ-5986 | TCGA-A3-3326 | TCGA-A3-3373 |
| TCGA-B0-5697 | TCGA-CJ-5679 | TCGA-CZ-5452 | TCGA-CZ-5987 | TCGA-A3-3328 | TCGA-A3-3374 |
| TCGA-B0-5699 | TCGA-CJ-5680 | TCGA-CZ-5453 | TCGA-CZ-5988 | TCGA-A3-3329 | TCGA-A3-3376 |
| TCGA-B0-5701 | TCGA-CJ-5681 | TCGA-CZ-5454 | TCGA-CZ-5989 | TCGA-A3-3331 | TCGA-A3-3378 |
| TCGA-B0-5703 | TCGA-CJ-5689 | TCGA-CZ-5455 | TCGA-3Z-A93Z | TCGA-A3-3335 | TCGA-A3-3380 |
| TCGA-B0-5705 | TCGA-CJ-6030 | TCGA-CZ-5456 | TCGA-6D-AA2E | TCGA-A3-3343 | TCGA-A3-3382 |
| TCGA-B0-5706 | TCGA-CJ-6033 | TCGA-CZ-5457 | TCGA-A3-3306 | TCGA-A3-3346 | TCGA-A3-3383 |
| TCGA-B0-5709 | TCGA-CW-5580 | TCGA-CZ-5458 | TCGA-A3-3307 | TCGA-A3-3347 | TCGA-A3-3385 |
| TCGA-B0-5711 | TCGA-CW-5581 | TCGA-CZ-5461 | TCGA-A3-3308 | TCGA-A3-3349 | TCGA-A3-3387 |
| TCGA-B0-5712 | TCGA-CW-5584 | TCGA-CZ-5462 | TCGA-A3-3311 | TCGA-A3-3351 | TCGA-A3-A6NI |
| TCGA-B2-5636 | TCGA-CW-5585 | TCGA-CZ-5463 | TCGA-A3-3313 | TCGA-A3-3352 | TCGA-A3-A6NJ |
| TCGA-B2-5641 | TCGA-CW-5587 | TCGA-CZ-5465 | TCGA-A3-3316 | TCGA-A3-3357 | TCGA-A3-A6NL |
| TCGA-A3-3358 | TCGA-B8-4619 | TCGA-CW-5589 | TCGA-CZ-5466 | TCGA-A3-3358 | TCGA-A3-A6NN |
| TCGA-B0-5107 | TCGA-B0-5075 | TCGA-B0-4824 | \| TCGA-B0-4703 \| \| --- \| | TCGA-AK-3454 | TCGA-A3-A8OU |
| TCGA-B0-5108 | TCGA-B0-5077 | TCGA-B0-4827 | TCGA-B0-4706 | TCGA-AK-3455 | TCGA-A3-A8OV |
| TCGA-B0-5109 | TCGA-B0-5080 | TCGA-B0-4828 | TCGA-B0-4707 | TCGA-AK-3456 | TCGA-A3-A8OW |
| TCGA-B0-5110 | TCGA-B0-5081 | TCGA-B0-4833 | TCGA-B0-4710 | TCGA-AK-3458 | TCGA-A3-A8OX |
| TCGA-B0-5113 | TCGA-B0-5083 | TCGA-B0-4834 | TCGA-B0-4712 | TCGA-AK-3460 | TCGA-AK-3425 |
| TCGA-B0-5115 | TCGA-B0-5084 | TCGA-B0-4836 | TCGA-B0-4713 | TCGA-AK-3461 | TCGA-AK-3426 |
| TCGA-B0-5116 | TCGA-B0-5085 | TCGA-B0-4837 | TCGA-B0-4714 | TCGA-AK-3465 | TCGA-AK-3427 |
| TCGA-B0-5117 | TCGA-B0-5088 | TCGA-B0-4838 | TCGA-B0-4718 | TCGA-AS-3777 | TCGA-AK-3428 |
| TCGA-B0-5119 | TCGA-B0-5092 | TCGA-B0-4839 | TCGA-B0-4810 | TCGA-AS-3778 | TCGA-AK-3429 |
| TCGA-B0-5120 | TCGA-B0-5094 | TCGA-B0-4841 | TCGA-B0-4811 | TCGA-B0-4688 | TCGA-AK-3431 |
| TCGA-B0-5121 | TCGA-B0-5095 | TCGA-B0-4842 | TCGA-B0-4813 | TCGA-B0-4690 | TCGA-AK-3433 |
| TCGA-B0-5399 | TCGA-B0-5096 | TCGA-B0-4843 | TCGA-B0-4814 | TCGA-B0-4691 | TCGA-AK-3434 |
| TCGA-B0-5400 | TCGA-B0-5097 | TCGA-B0-4844 | TCGA-B0-4815 | TCGA-B0-4693 | TCGA-AK-3436 |
| TCGA-B0-5402 | TCGA-B0-5098 | TCGA-B0-4845 | TCGA-B0-4816 | TCGA-B0-4694 | TCGA-AK-3440 |
| TCGA-B0-5690 | TCGA-B0-5099 | TCGA-B0-4846 | TCGA-B0-4817 | TCGA-B0-4696 | TCGA-AK-3443 |
| TCGA-B0-5691 | TCGA-B0-5100 | TCGA-B0-4847 | TCGA-B0-4818 | TCGA-B0-4697 | TCGA-AK-3445 |
| TCGA-B0-5692 | TCGA-B0-5102 | TCGA-B0-4848 | TCGA-B0-4819 | TCGA-B0-4698 | TCGA-AK-3447 |
| TCGA-B0-5693 | TCGA-B0-5104 | TCGA-B0-4849 | TCGA-B0-4821 | TCGA-B0-4699 | TCGA-AK-3450 |
| TCGA-B0-5694 | TCGA-B0-5106 | TCGA-B0-4852 | TCGA-B0-4822 | TCGA-B0-4700 | TCGA-AK-3451 |
| TCGA-B0-5695 | TCGA-B0-4945 | TCGA-B0-4823 | TCGA-B0-4701 | TCGA-AK-3453 | TCGA-A3-A8CQ |
| TCGA-B0-5107 | TCGA-B0-5696 | TCGA-B2-3924 | TCGA-B4-5834 | TCGA-BP-4158 | TCGA-BP-4330 |
| TCGA-B0-5697 | TCGA-B2-3924 | TCGA-B4-5835 | TCGA-B8-5164 | TCGA-BP-4159 | TCGA-BP-4331 |
| TCGA-B0-5698 | TCGA-B2-3924 | TCGA-B4-5836 | TCGA-B8-5165 | TCGA-BP-4160 | TCGA-BP-4332 |
| TCGA-B0-5699 | TCGA-B2-4098 | TCGA-B4-5838 | TCGA-B8-5545 | TCGA-BP-4161 | TCGA-BP-4334 |
| TCGA-B0-5700 | TCGA-B2-4099 | TCGA-B4-5843 | TCGA-B8-5546 | TCGA-BP-4162 | TCGA-BP-4335 |
| TCGA-B0-5701 | TCGA-B2-4101 | TCGA-B4-5844 | TCGA-B8-5549 | TCGA-BP-4163 | TCGA-BP-4337 |
| TCGA-B0-5702 | TCGA-B2-4102 | TCGA-B8-4143 | TCGA-B8-5550 | TCGA-BP-4164 | TCGA-BP-4338 |
| TCGA-B0-5703 | TCGA-B2-5633 | TCGA-B8-4146 | TCGA-B8-5551 | TCGA-BP-4165 | TCGA-BP-4340 |
| TCGA-B0-5705 | TCGA-B2-5633 | TCGA-B8-4148 | TCGA-B8-5552 | TCGA-BP-4166 | TCGA-BP-4341 |
| TCGA-B0-5706 | TCGA-B2-5633 | TCGA-B8-4151 | TCGA-B8-5553 | TCGA-BP-4167 | TCGA-BP-4342 |
| TCGA-B0-5707 | TCGA-B2-5635 | TCGA-B8-4153 | TCGA-B8-A54D | TCGA-BP-4169 | TCGA-BP-4343 |
| TCGA-B0-5709 | TCGA-B2-5635 | TCGA-B8-4154 | TCGA-B8-A54E | TCGA-BP-4170 | TCGA-BP-4344 |
| TCGA-B0-5710 | TCGA-B2-5635 | TCGA-B8-4619 | TCGA-B8-A54F | TCGA-BP-4173 | TCGA-BP-4345 |
| TCGA-B0-5711 | TCGA-B2-5636 | TCGA-B8-4620 | TCGA-B8-A54G | TCGA-BP-4174 | TCGA-BP-4346 |
| TCGA-B0-5712 | TCGA-B2-5639 | TCGA-B8-4621 | TCGA-B8-A54H | TCGA-BP-4176 | TCGA-BP-4347 |
| TCGA-B0-5713 | TCGA-B2-5641 | TCGA-B8-4622 | TCGA-B8-A54I | TCGA-BP-4177 | TCGA-BP-4349 |
| TCGA-B0-5812 | TCGA-B2-A4SR | TCGA-B8-5158 | TCGA-B8-A54J | TCGA-BP-4325 | TCGA-BP-4351 |
| TCGA-B2-3923 | TCGA-B4-5377 | TCGA-B8-5159 | TCGA-B8-A54K | TCGA-BP-4326 | TCGA-BP-4352 |
| TCGA-B2-3923 | TCGA-B4-5378 | TCGA-B8-5162 | TCGA-B8-A7U6 | TCGA-BP-4327 | TCGA-BP-4353 |
| TCGA-B2-3923 | TCGA-B4-5832 | TCGA-B8-5163 | TCGA-B8-A8YJ | TCGA-BP-4329 | TCGA-BP-4354 |
| TCGA-BP-4756 | TCGA-BP-4787 | TCGA-BP-4969 | TCGA-BP-4995 | TCGA-BP-5180 | TCGA-CJ-4634 |
| TCGA-BP-4758 | TCGA-BP-4789 | TCGA-BP-4970 | TCGA-BP-4998 | TCGA-BP-5181 | TCGA-CJ-4635 |
| TCGA-BP-4759 | TCGA-BP-4790 | TCGA-BP-4971 | TCGA-BP-4999 | TCGA-BP-5182 | TCGA-CJ-4636 |
| TCGA-BP-4760 | TCGA-BP-4795 | TCGA-BP-4972 | TCGA-BP-5000 | TCGA-BP-5183 | TCGA-CJ-4637 |
| TCGA-BP-4761 | TCGA-BP-4797 | TCGA-BP-4973 | TCGA-BP-5001 | TCGA-BP-5184 | TCGA-CJ-4638 |
| TCGA-BP-4762 | TCGA-BP-4798 | TCGA-BP-4974 | TCGA-BP-5004 | TCGA-BP-5185 | TCGA-CJ-4639 |
| TCGA-BP-4763 | TCGA-BP-4799 | TCGA-BP-4975 | TCGA-BP-5006 | TCGA-BP-5186 | TCGA-CJ-4640 |
| TCGA-BP-4765 | TCGA-BP-4801 | TCGA-BP-4976 | TCGA-BP-5007 | TCGA-BP-5187 | TCGA-CJ-4641 |
| TCGA-BP-4766 | TCGA-BP-4803 | TCGA-BP-4977 | TCGA-BP-5008 | TCGA-BP-5189 | TCGA-CJ-4642 |
| TCGA-BP-4768 | TCGA-BP-4804 | TCGA-BP-4981 | TCGA-BP-5009 | TCGA-BP-5190 | TCGA-CJ-4643 |
| TCGA-BP-4769 | TCGA-BP-4807 | TCGA-BP-4982 | TCGA-BP-5010 | TCGA-BP-5191 | TCGA-CJ-4644 |
| TCGA-BP-4770 | TCGA-BP-4959 | TCGA-BP-4983 | TCGA-BP-5168 | TCGA-BP-5192 | TCGA-CJ-4868 |
| TCGA-BP-4771 | TCGA-BP-4960 | TCGA-BP-4985 | TCGA-BP-5169 | TCGA-BP-5194 | TCGA-CJ-4869 |
| TCGA-BP-4774 | TCGA-BP-4961 | TCGA-BP-4986 | TCGA-BP-5170 | TCGA-BP-5195 | TCGA-CJ-4870 |
| TCGA-BP-4775 | TCGA-BP-4962 | TCGA-BP-4987 | TCGA-BP-5173 | TCGA-BP-5196 | TCGA-CJ-4871 |
| TCGA-BP-4776 | TCGA-BP-4963 | TCGA-BP-4989 | TCGA-BP-5174 | TCGA-BP-5198 | TCGA-CJ-4872 |
| TCGA-BP-4777 | TCGA-BP-4964 | TCGA-BP-4991 | TCGA-BP-5175 | TCGA-BP-5199 | TCGA-CJ-4873 |
| TCGA-BP-4781 | TCGA-BP-4965 | TCGA-BP-4992 | TCGA-BP-5176 | TCGA-BP-5200 | TCGA-CJ-4874 |
| TCGA-BP-4782 | TCGA-BP-4967 | TCGA-BP-4993 | TCGA-BP-5177 | TCGA-BP-5201 | TCGA-CJ-4875 |
| TCGA-BP-4355 | TCGA-BP-4784 | TCGA-BP-4968 | TCGA-BP-4994 | TCGA-BP-5178 | TCGA-BP-5202 |
| TCGA-CJ-4878 | TCGA-CJ-4902 | TCGA-CJ-5684 | TCGA-CW-6088 | TCGA-CZ-5454 | TCGA-CZ-5985 |
| TCGA-CJ-4881 | TCGA-CJ-5682 | TCGA-CJ-5686 | TCGA-CW-6090 | TCGA-CZ-5455 | TCGA-CZ-5986 |
| TCGA-CJ-4882 | TCGA-CJ-4903 | TCGA-CJ-5689 | TCGA-CW-6093 | TCGA-CZ-5456 | TCGA-CZ-5987 |
| TCGA-CJ-4884 | TCGA-CJ-4904 | TCGA-CJ-6027 | TCGA-CW-6097 | TCGA-CZ-5457 | TCGA-CZ-5988 |
| TCGA-CJ-4885 | TCGA-CJ-4905 | TCGA-CJ-6028 | TCGA-CZ-4853 | TCGA-CZ-5458 | TCGA-CZ-5989 |
| TCGA-CJ-4886 | TCGA-CJ-4907 | TCGA-CJ-6030 | TCGA-CZ-4854 | TCGA-CZ-5459 | TCGA-DV-5565 |
| TCGA-CJ-4887 | TCGA-CJ-4908 | TCGA-CJ-6031 | TCGA-CZ-4856 | TCGA-CZ-5460 | TCGA-DV-5566 |
| TCGA-CJ-4888 | TCGA-CJ-4912 | TCGA-CJ-6032 | TCGA-CZ-4857 | TCGA-CZ-5461 | TCGA-DV-5567 |
| TCGA-CJ-4889 | TCGA-CJ-4916 | TCGA-CJ-6033 | TCGA-CZ-4858 | TCGA-CZ-5462 | TCGA-DV-5568 |
| TCGA-CJ-4890 | TCGA-CJ-4918 | TCGA-CW-5580 | TCGA-CZ-4859 | TCGA-CZ-5463 | TCGA-DV-5569 |
| TCGA-CJ-4891 | TCGA-CJ-4920 | TCGA-CW-5581 | TCGA-CZ-4860 | TCGA-CZ-5464 | TCGA-DV-5573 |
| TCGA-CJ-4892 | TCGA-CJ-5671 | TCGA-CW-5583 | TCGA-CZ-4861 | TCGA-CZ-5465 | TCGA-DV-5574 |
| TCGA-CJ-4893 | TCGA-CJ-5672 | TCGA-CW-5584 | TCGA-CZ-4862 | TCGA-CZ-5466 | TCGA-DV-5575 |
| TCGA-CJ-4894 | TCGA-CJ-5675 | TCGA-CW-5585 | TCGA-CZ-4863 | TCGA-CZ-5467 | TCGA-DV-5576 |
| TCGA-CJ-4895 | TCGA-CJ-5676 | TCGA-CW-5587 | TCGA-CZ-4864 | TCGA-CZ-5468 | TCGA-DV-A4VX |
| TCGA-CJ-4897 | TCGA-CJ-5677 | TCGA-CW-5588 | TCGA-CZ-4865 | TCGA-CZ-5469 | TCGA-DV-A4VZ |
| TCGA-CJ-4899 | TCGA-CJ-5678 | TCGA-CW-5589 | TCGA-CZ-4866 | TCGA-CZ-5470 | TCGA-DV-A4W0 |
| TCGA-CJ-4900 | TCGA-CJ-5679 | TCGA-CW-5590 | TCGA-CZ-5451 | TCGA-CZ-5982 | TCGA-DV-A4W0 |
| TCGA-CJ-4901 | TCGA-CJ-5680 | TCGA-CW-5591 | TCGA-CZ-5452 | TCGA-CZ-5984 | TCGA-EU-5904 |
| TCGA-CJ-4876 | TCGA-CJ-5681 | TCGA-CJ-5683 | TCGA-CW-6087 | TCGA-CZ-5453 | TCGA-EU-5905 |
| TCGA-EU-5907 | TCGA-G6-A5PC | TCGA-G6-A8L6 | TCGA-G6-A8L7 | TCGA-G6-A8L8 | TCGA-T7-A92I |
| TCGA-MW-A4EC | TCGA-MM-A84U | TCGA-MM-A564 | TCGA-MM-A563 | TCGA-GK-A6C7 |  |
